# Genomic risk profiling in advanced maternal age: a Tamil Nadu prenatal study

**DOI:** 10.3389/fmed.2026.1864106

**Published:** 2026-08-28

**Authors:** Sujithra Appavu, Aruni Wilson Santhosh Kumar, Mukinkumar Sonai, Vinoth Appavu

**Affiliations:** 1 Department of Biotechnology, Sathyabama Institute of Science and Technology, Chennai, Tamil Nadu, India; 2 Department of Cytogenetics, Life Cell International Private Ltd., Chennai, Tamil Nadu, India; 3 Amity University, Mumbai, Maharashtra, India; 4 Department of Biologics, Life Cell International Private Ltd., Chennai, Tamil Nadu, India; 5 Department of Emergency Medicine, Thanjavur Medical College, Thanjavur, Tamil Nadu, India

**Keywords:** advanced maternal age, aneuploidy, chromosomal microarray, genetic abnormalities, India, karyotyping, prenatal diagnosis

## Abstract

**Background:**

Advanced maternal age (AMA; > = 35 years) is associated with increased fetal chromosomal risk and is an important indication for invasive prenatal diagnosis. This study evaluates karyotyping and chromosomal microarray analysis (CMA) findings among AMA pregnancies in Tamil Nadu, India.

**Methods:**

In this prospective observational study, 2,200 pregnant women aged ≥35 years who underwent amniocentesis between July 2020 and June 2021 were enrolled. Conventional karyotyping was performed in all cases. CMA was performed as a reflex or complementary test when predefined clinical or cytogenetic criteria were present, including ultrasound abnormalities, high-risk screening/NIPT results, abnormal or uncertain karyotype findings, prior adverse pregnancy history suggestive of genetic etiology, parental chromosomal abnormalities, or patient request after counseling.

**Results:**

Numerical chromosomal abnormalities were detected in 76/2200 cases (3.5%), and structural abnormalities were detected in 9/2200 cases (0.4%). The total abnormal cytogenetic yield was therefore 85/2200 cases (3.9%) when numerical and structural abnormalities were considered together. Age-stratified analysis showed an increasing trend in chromosomal abnormalities with advancing maternal age, reaching 10.4% in women aged > = 43 years. Cases with additional clinical indications showed higher abnormality rates than simple AMA. CMA was performed in selected cases and contributed to characterization of clinically relevant genomic abnormalities, supporting its role in indicated high-risk AMA pregnancies.

**Conclusion:**

The Tamil Nadu cohort provides regional evidence on age-stratified cytogenetic risk in AMA pregnancies and supports the use of clearly defined criteria for integrating CMA with conventional karyotyping in prenatal diagnosis.

## 1 Introduction

Recent years have witnessed a massive global demographic transition, resulting in a steady rise in maternal age at conception. This is largely due to sociocultural changes, higher educational attainment, delayed marriage, and career priorities (1, 2). Advanced maternal age (AMA) in general can be defined as the occurrence of pregnancy among women who are aged 35 years and above, consistently found to be in association with increased risks of adverse fetal and maternal outcomes (3). Major outcomes were reduced fertility rates and higher rates of stillbirths, abortion, and chromosomal abnormalities (trisomy 18, trisomy 21, and trisomy 13) (4–6). AMA was strongly correlated with genetic anomalies that contribute for early pregnancy loss, posing a significant challenge within reproductive health systems (7). Major neonatal complications in AMA are low Apgar score, preterm delivery, higher likelihood of NICU admission, low birth weight, chromosomal abnormalities, birth defects, and perinatal death (6, 8, 9).

In India, resultant urbanizations, sociocultural changes, and economic growth are main factors resulting in increased maternal cases conceiving at later ages (10, 11). Traditionally, standard karyotype analysis served as the cornerstone for prenatal genetic diagnosis, detecting large-scale chromosomal abnormalities, such as aneuploidies, structural aberrations, and unbalanced rearrangements (8). In recent years, prenatal analysis via chromosomal microarray analysis (CMA) emerged as a high-resolution technique that is capable of identifying clinically significant copy number variations (CNVs) that remain undetectable in conventional karyotyping (12, 13). Studies demonstrated that CMA can still uncover pathogenic or potentially pathogenic genomic imbalances in those cases that were reported normal in karyotyping, especially in AMA pregnancies (14). This improves overall detection rates but also provides clinicians and genetic counselors with robust information for expectant parents (15). The present article provides regional, age-stratified cytogenetic evidence from Tamil Nadu and describes a practical diagnostic workflow combining karyotyping and CMA in AMA pregnancies.

## 2 Methods

### 2.1 Patients and design

This prospective observational study involved about 2,200 pregnant women aged 35 years or older who had an amniocentesis for prenatal diagnosis between July 2020 and June 2021 at LifeCell International Private Limited, Chennai, Tamil Nadu, India. All participants received pretest genetic counseling, and they also gave written informed consent. The gestational age at the time of enrolment ranged between 15 and 25 weeks. The whole study protocol was reviewed and approved by the Institutional Review Board and Institutional Biosafety and Ethical Committee (Ref: 137/IRB-IBSEC/SIST, dated 19 November 2019).

#### 2.1.1 Definition of referral categories and analytical groups

To clarify the grouping logic, two non-identical classification steps were employed in this study. The label “AMA only” denotes the primary referral indication recorded at enrollment and is used for the baseline descriptive comparison in Table 1 (*n* = 1,530). By contrast, “simple AMA” denotes the final chart-reviewed analytic risk group used for the diagnostic-yield comparison in Table 2. Pregnancies were classified as AMA combined with other indications only when at least one confirmed additional high-risk clinical or genetic indication was documented; cases without such confirmed additional indications were retained in the simple AMA analytic group. This rule explains the different denominators: the simple AMA analytic group contains 1958 pregnancies, including the 1,530 primary AMA-only referrals plus 428 cases that did not meet the final confirmed additional-risk definition. The remaining 242 pregnancies were classified as AMA combined with other indications.

**Table 1:** Comparison of maternal age (years) and gestational age (weeks) across primary referral indication.

Variable	Subgroup	*N*	Mean	SD	95% CI	ANOVA (between groups)	df	Mean square	*F*	*p*-value
					(LB–UB)					
Gestational Age (weeks)	AMA only	1,530	17.84	2.11	17.74–17.95					
	ART	29	18.76	2.42	17.84–19.68					
	Biochemical screen positive	148	17.73	2.18	17.37–18.08					
	High-risk NIPT	81	18.31	2.35	17.79–18.83					
	History of adverse pregnancy	64	18.36	2.3	17.79–18.93	SS = 59.576	6,2193	9.929	2.183	0.042*
	Twins	24	17.58	2.5	16.53–18.64					
	Ultrasound abnormality	324	17.88	2.05	17.66–18.11					
	Total	2,200	17.88	2.14	17.79–17.97					
Maternal Age (years)	AMA only	1,530	37.22	1.86	37.13–37.32					
	ART	29	37	1.87	36.29–37.71					
	Biochemical screen Positive	148	37.41	1.89	37.11–37.72					
	High-risk NIPT	81	37.75	2	37.31–38.20					
	History of adverse pregnancy	64	37.45	1.77	37.01–37.90	SS = 45.016	6,2193	7.503	2.146	0.045*
	Twins	24	38.04	1.85	37.26–38.82					
	Ultrasound abnormality	324	37.35	1.87	37.14–37.55					
	Total	2,200	37.29	1.87	37.21–37.36					

AMA, advanced maternal age; ART, assisted reproductive technology; NIPT, non-invasive prenatal testing; SD, standard deviation; CI, confidence interval; ANOVA, analysis of variance.

Values are presented as mean ± standard deviation (SD) and 95% confidence intervals (CI). One-way ANOVA was used to compare means across groups. A *p*-value < 0.05 was considered statistically significant. The “AMA only” subgroup in table is the referral-indication category of the study sample (*n* = 1,530 enrolments). This subgroup refers only to baseline descriptive comparisons, and the subgroup reported as final “AMA Simple” in Table 2 is not synonymous with this subgroup. The symbol “*” denotes statistical significance (*p* < 0.05).

**Table 2:** Distribution of cytogenetic abnormalities among pregnancies with advanced maternal age.

Group	*N*	Chromosomal abnormalities		Chi-square (df)	*p*-value
		Number of abnormalities (%)	Structural abnormalities (%)		
Simple AMA	1958	53 (2.7)	4 (0.2)	18.006 (1)	0.000*
AMA combined with other indications	242	19 (7.9)	5 (2.1)	18.325 (1)	0.000*
Total	2,200	72 (3.3)	9 (0.4)		

AMA, advanced maternal age; *χ*^2^, Chi-square statistic.

Simple AMA refers to pregnancies referred solely because of advanced maternal age. AMA with additional indications refers to pregnancies presenting with one or more additional clinical risk factors, including abnormal ultrasound findings, positive prenatal screening results, previous adverse pregnancy history, assisted reproductive technology, or other high-risk indications. “Simple AMA” column includes the chart-reviewed analytic group of all high-risk pregnancies that do not have a verified high-risk clinical/ genetic indication (*n* = 1968). This analytic group includes all of the pregnancy cases in the AMA and AMA referral categories and is different from “AMA only,” which was presented in the primary referral category (*n* = 1,530). The difference of 428 pregnancies between these two analytic groups results from pregnancies that were originally recorded outside of the primary AMA only category for baseline information and were subsequently confirmed to have additional high-risk indications once the charts were reviewed. As a result, these pregnancies remain in the simple AMA group. The “AMA combined with other indications” category includes any pregnancies that contain a minimum of one additional responsible indication (*n* = 242). The symbol “*” denotes statistical significance (*p* < 0.05).

### 2.2 Prenatal diagnosis

Amniocentesis (AC) was performed under ultrasound guidance for conducting cytogenetic analysis. Cell cultures were established and maintained according to the manufacturer’s protocols using commercially available amniotic fluid culture media. Cell culture procedures were carried out in an Esco Class II biosafety cabinet (Esco Lifesciences, Singapore), and cultures were incubated in a CellXpert^®^ CO₂ incubator (Eppendorf AG, Hamburg, Germany) under controlled conditions. Chromosomal abnormalities and structural rearrangements were classified following ISCN 2020 standards.

### 2.3 CMA analysis

Approximately 10 mL of amniotic fluid was collected, and genomic DNA was extracted using QIAamp DNA Mini Kit (Qiagen, Valencia, CA, United States). DNA samples were amplified, labeled, and hybridized to the Affymetrix CytoScan 750 K Array platform according to manufacturer’s instructions. Following hybridization, arrays were scanned using a GCS 3000Dx v.2 system (Affymetrix, United States). Data were processed with Chromosome Analysis Suite (ChAS) software (version 3.2, Thermo Fisher Scientific). Submicroscopic CNVs and other genomic imbalances were interpreted with reference to established databases, including DECIPHER, ClinVar, and the Database of Genomic Variants.

Chromosomal microarray analysis (CMA) was performed in this study as a reflex test for pregnancy cases that required additional high-risk findings, such as abnormal ultrasound findings of the fetus, positive prenatal screening outcomes, suspected chromosomal abnormalities that require further evaluation, or when recommended during genetic counseling. Pregnancies with AMA as sole indication were generally subjected to conventional karyotyping, while CMA was selectively carried out among cases with additional clinical risk factors.

## 3 Statistical analysis

All statistical analyses were conducted using SPSS software version 23.0 for Windows; (IBM Corp., Armonk, NY, United States). Continuous variables were summarized as mean +/− standard deviation (SD) and 95% confidence intervals (CIs). Categorical variables were summarized as counts and percentages. One-way ANOVA was used to compare maternal age and gestational age across referral groups; when global tests were significant, *post hoc* pairwise comparisons with Bonferroni correction were planned. Data comparisons between categories were performed with chi-square tests, and when the expected cell count was less than 5, the Fisher Exact test was utilized. The Cochran-Armitage Trend Test was performed to evaluate chromosomal abnormalities by age. Effect size estimates of the results will be reported in odds ratios (OR), with corresponding 95% confidence intervals (CIs). Multivariable logistic regression analyses were planned to investigate the association between chromosomal abnormality and maternal age and controlled for multiple additional factors including: indication status, gestational age, assisted reproductive technology (ART) conception, ultrasound abnormality status, and high-risk screening/non-invasive prenatal testing (NIPT) method. A *p*-value less than or equal to 0.05 was considered statistically significant; however, for *p*-values less than or equal to 0.001, the result will be reported as *p* < 0.001 instead of *p* = 0.000.

## 4 Results

### 4.1 Study population and baseline characteristics

During the unified study period from July 2020 to June 2021, 2,200 AMA pregnancies underwent amniocentesis for prenatal diagnosis. The mean gestational age at testing was 17.88 +/2.14 weeks, and the mean maternal age was 37.29 +/−1.87 years. This value shows only a small amount of change when the data is stratified by indication. The largest subgroup was women referred for AMA only (*n* = 1,530), with a mean gestational age of 17.84 ± 2.11 weeks (95% CI: 17.74–17.95). For the cases undergoing testing due to ART (*n* = 29), these cases had the highest mean gestational age (18.76 ± 2.42 weeks; 95% CI: 17.84–19.68). Likewise, referrals after high-risk NIPT result (*n* = 81) and those with a prior history of adverse pregnancy outcome (*n* = 64) showed elevated mean gestational ages of 18.31 ± 2.35 and 18.36 ± 2.30, respectively.

AMA women with biochemical screening presented with positive results (*n* = 148), and those with ultrasound abnormalities (*n* = 324) had similar mean ages of 17.73 ± 2.18 weeks and 17.88 ± 2.05 weeks, respectively. The small subgroup of twin pregnancies (*n* = 24) had the lowest mean advanced gestational age (17.58 ± 2.50 weeks; 95% CI: 16.53–18.64).

One-way ANOVA analysis revealed a significant difference in maternal age across the seven groups [*F*(6, 2,193) = 2.146, *p* = 0.045]. The analysis revealed a significant effect of referral indication upon gestational age: [*F* (6, 2,193) = 2.183, *p* = 0.042]. Descriptive statistics revealed that mean ages of mothers in the study ranged from 37.00 ± 1.87 years in the ART group to 38.04 ± 1.85 years in the Twins group. The largest subgroup, AMA only\-category (*n* = 1,530), resulted in the mean maternal age of 37.22 ± 1.86 years, which closely followed the cohort average mean maternal age of 37.29 ± 1.87 years.

Subgroups, such as biochemical screen positive (37.41 ± 1.89 years), high-risk NIPT (37.75 ± 2.00 years), and an adverse outcome pregnancy history (37.45 ± 1.77 years), demonstrated elevated mean ages in comparison to the AMA only group. The most notable finding was the Twins subgroup with the highest mean maternal age of 38.04 years, although the result was from a small sample size (*n* = 24). The baseline category “AMA only” in Table 1 therefore refers to the primary referral label at enrollment, whereas the “simple AMA” denominator used later for outcome analysis is a final chart-reviewed analytic group.

### 4.2 Diagnostic yield according to analytic indication group

For diagnostic yield analysis, participants were reconsolidated into two final analytic risk groups from review of clinical referral and cytogenetic records. Simple AMA was defined as AMA with no verified, additional, high-level clinical or genetic indication. A total of 1,958 pregnancies were present in this group, so this group was different from the “AMA only” baseline reference group (*n* = 1,530) for primary referral. The numerical differences were not the result of an error in the data; they are given in Table 1 (the primary referral label) and Table 2 (the final chart reviewed analytic grouping rule). Therefore, since 428 cases were initially recorded in the baseline description as being outside of the primary AMA only referral category but failed to meet the final confirmed additional risk definition, they were included in the simple AMA group. The rest of the pregnancies (242) had at least one confirmed additional indication and were included in the AMA and other indication analyses. The diagnostic yield values for each of the two analytic groups are presented in Table 2.

With that approach, numerical chromosomal abnormalities showed up in 57/1,958 of the simple AMA group (2.9%) and in 19/242 of the cases with additional indications (7.9%). Structural abnormalities were found in 4/1,958 simple AMA cases (0.2%) and 5/242 cases where there were additional indications (2.1%). Additional indication status lines up with higher odds for numerical chromosomal abnormality (OR 2.84, 95% CI 1.66–4.86, chi-square = 15.760, *p* < 0.001) and also for structural abnormality (OR 10.31; 95% CI 2.75–38.65; chi-square = 18.325; *p* < 0.001).

### 4.3 Abnormal findings among AMA pregnancies with additional indications

In among 242 AMA pregnancies with additional indications, a history of adverse pregnancy was the largest subgroup (*n* = 63, 26.0%), then came high-risk prenatal screening (*n* = 43, 17.8%), ART conception (*n* = 41, 16.9%), twin pregnancy (*n* = 33, 13.6%), ultrasonographic abnormalities (*n* = 31, 12.8%), spouse chromosomal abnormalities (*n* = 29, 12.0%), and other indications (*n* = 2, 0.8%).

### 4.4 Diagnostic findings by test modality

Conventional karyotyping was done in all the cases. CMA was then applied in selected cases based on the criteria that are laid out in the Methods section. In the whole cohort, 2,116/2,200 cases were said to be cytogenetically normal by whatever test modality was used. The spread of diagnostic categories by each test modality is summarized in Table 3. Since CMA was not carried out on every single sample, this dataset should not be taken as showing a universal extra CMA yield over karyotyping; instead, it basically illustrates the diagnostic contribution of CMA within those clinically indicated situations.

**Table 3:** Distribution of cytogenetic findings among AMA pregnancies with additional clinical indications.

Clinical indications	*n* (%)	Numerical abnormalities	Structural abnormalities
ART	41 (16.9)	5	0
High risk of prenatal screening	43 (17.8)	4	1
History of adverse pregnancy	63 (26.0)	3	0
Spouse chromosome abnormalities	29 (12.0)	2	2
Ultrasonographic abnormalities	31 (12.8)	2	1
Twins	33 (13.6)	3	1
Others	2 (0.8)	0	0

AMA, advanced maternal age; ART, assisted reproductive technology.

Note: Percentages are calculated based on the subgroup of pregnancies with AMA and additional clinical indications (*n* = 242).

The Pearson chi-square for how the diagnostic categories were spread across the different test modalities came out as chi-square = 25.575, df = 14, *p* = 0.029. This kind of comparison basically points to variation in the diagnostic category distribution but not really the kind of paired incremental-yield analysis.

### 4.5 Age-specific distribution of chromosomal abnormalities

In the age-stratified analysis, 76 numerical chromosomal abnormalities (3.5%) and 9 structural abnormalities were identified (0.4%). The earlier, invalid structural figure of 0.5 for age 41 was then fixed back to 0 because counts really have to be whole numbers. For numerical chromosomal problems, the rates went up as maternal age increased, climbing to 10.4% in women aged ≥ 43 years (Tables 4, 5).

**Table 4:** Prenatal diagnosis in the AMA women who selected both karyotype analysis and CMA.

Cytogenetic finding		Test modality			Total	Pearson Chi-square (df)	*p*-value
		CMA only	Combined karyotype + CMA	Standard karyotype only			
Karyotype Detail	Normal Karyotype (46, XX and 46, XY)	265	606	1,245	2,116	25.575 (14)	0.029
	Inversion/duplication	0	5	7	12		
	Marker chromosome	0	2	10	12		
	Monosomy X	0	3	14	17		
	Trisomy 13	0	10	5	15		
	Trisomy 18	0	2	2	4		
	Trisomy 21	1	7	7	15		
	Unbalanced translocation	1	4	4	9		
Total		267	639	1,294	2,200		

CMA, chromosomal microarray analysis; χ^2^, Chi-square statistic. Pearson’s chi-square test was used to compare cytogenetic findings among testing modalities.

**Table 5:** Maternal age-specific distribution of chromosomal abnormalities in advanced maternal age pregnancies.

Maternal age (in years)	*n* (%)	Number of abnormalities, *n* (%)	Structural abnormalities, *n* (%)
	(Total = 2,200)		
35	504 (22.9%)	14 (2.7%)	3 (0.6%)
36	466 (21.2%)	8 (1.8%)	1 (0.2%)
37	358 (16.3%)	8 (2.3%)	3 (0.9%)
38	281 (12.8%)	17 (5.9%)	0 (0%)
39	201 (9.1%)	4 (1.8%)	1 (0.5%)
40	154 (7.0%)	8 (5.4%)	0 (0%)
41	107 (4.9%)	8 (7.3%)	0.5 (0.5%)
42	62 (2.8%)	2 (3.4%)	1 (1.7%)
≥43	67 (3.0%)	7 (10.2%)	0 (0%)
Total	2,200 (100%)	76 (3.4%)	9.5 (0.4%)

AMA, advanced maternal age. Numerical abnormalities include aneuploidies and other numerical chromosomal abnormalities. Structural abnormalities include chromosomal rearrangements and structural cytogenetic abnormalities detected during prenatal testing.

### 4.6 Contribution and limitations of CMA in selected cases

The CMA was used selectively rather than in a blanket way. The study no longer stands by an unsupported 2.5% universal incremental diagnostic yield, which is good. Instead, the findings show that CMA can deliver clinically useful genomic characterization in those indicated high-risk AMA pregnancies, especially when ultrasound results, screening outcomes, uncertain karyotype signals, or even parental chromosomal abnormalities seem to point to why higher resolution testing is warranted.

**Figure 1:**
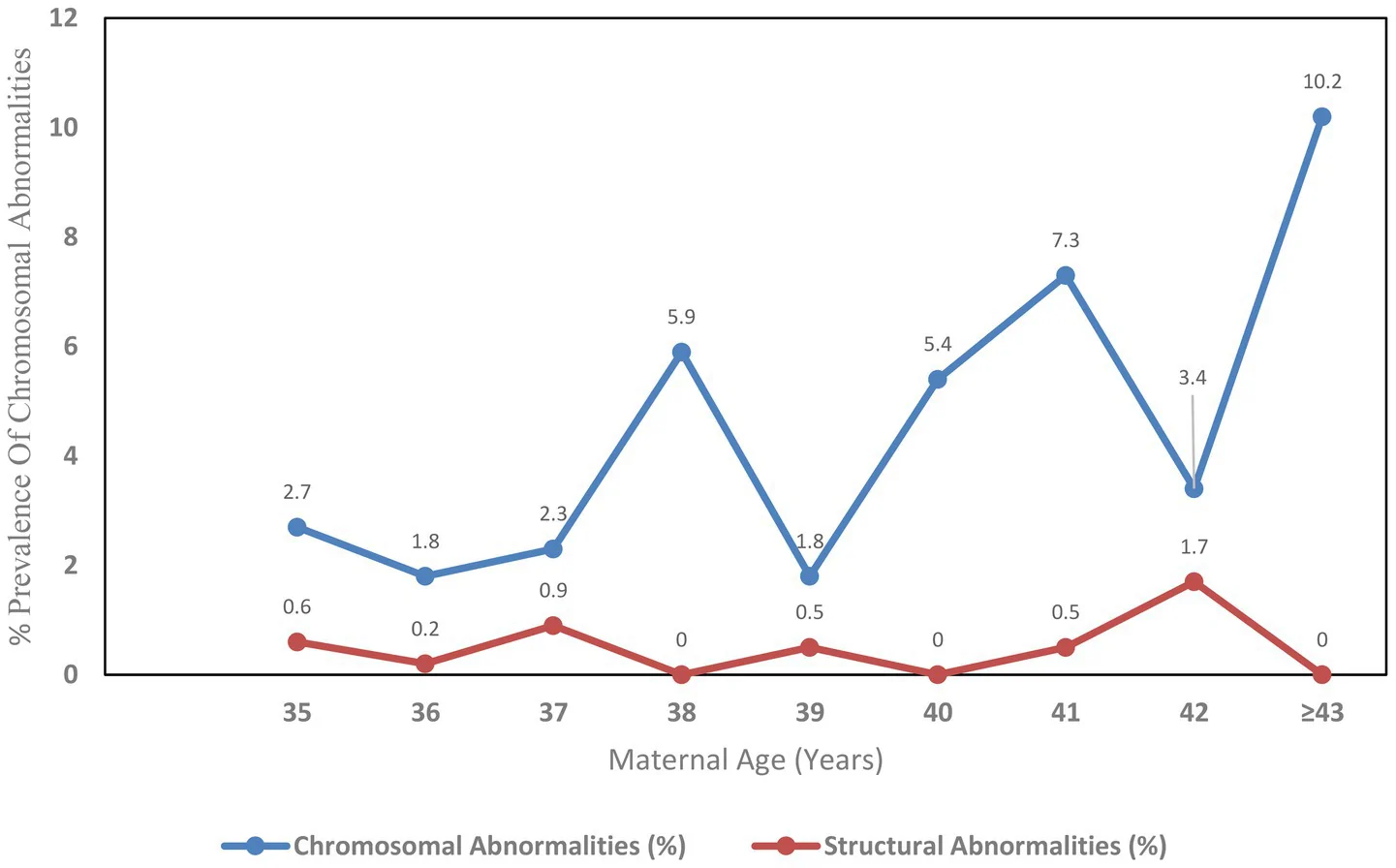
Maternal age and prevalence of chromosomal abnormalities.

Figure 1 shows maternal age and prevalence of chromosomal abnormalities. The investigation disclosed a pronounced age-related gradient in the occurrence of chromosomal abnormalities in AMA women. Prevalence was minimal at ages 36 and 39 (both 1.8%), but thereafter a steep gradient appeared, culminating at ≥43 years, where 10.2% of samples exhibited an abnormal karyotype.

## 5 Discussion

Our study evaluated chromosomal abnormalities among 2,200 AMA pregnancies subjected to invasive prenatal diagnosis as our primary focus toward the diagnostic contribution of conventional karyotyping and CMA. The principal findings involved assessing the overall prevalence of chromosomal abnormalities, reported in 8.1% of cases with trisomy 21, presenting as the most frequent anomaly observed from prenatal diagnosis. Conversely, an earlier cohort survey presented with a larger population screening showed 2.4% of the AMA cases with genetic abnormalities from karyotyping (16). The predominance of trisomy 21 in our cohort is consistent with epidemiological data demonstrating that Down syndrome remains the most frequent chromosomal abnormality among older pregnant women (17, 18). Another major finding involved with determining abnormalities related risks and their correlation with AMA. Moreover, the steep rise in abnormality detection among women ≥40 years, with rates exceeding 15% in our study, is in accordance with prior studies documenting exponential risk escalation after this threshold (19). AMA combined with additional risk factors (such as abnormal ultrasound findings or positive biochemical screening) presented with a greater abnormality detection rate compared to AMA alone. Furthermore, CMA provided an incremental diagnostic yield of 2.5%, mainly through the identification of pathogenic CNVs that were undetectable via karyotyping. Also, the data revealed that AMA cases with other high-risk features had a significantly higher abnormality rate compared to those with AMA alone. This is in agreement with reports presented from ultrasound abnormalities or abnormal biochemical markers that substantially increased the probability of clinically significant cytogenetic findings (20, 21).

The incremental diagnostic yield of 2.5% observed in our series was in accordance with AMA-focused studies (15, 16), with 1–3% additional yield reported. Importantly, pathogenic CNVs identified were clinically significant and would have been missed by conventional karyotyping. This supports the recommendation of the American College of Medical Genetics (ACMG) that CMA should be considered as a first-tier test for high-risk pregnancies, particularly in ultrasound abnormalities (22).

However, there are certain limitations needed to be acknowledged. The single-center design may restrict generalizability, as patient populations and referral patterns vary regionally. In addition, CMA was not universally performed across all cases, introducing potential selection bias, as women with abnormal ultrasounds may have been more likely to undergo CMA. Moreover, the lack of long-term follow-up data limits our ability to correlate identified CNVs with postnatal phenotypic outcomes, particularly for variants of uncertain significance (VOUS). Finally, the absence of a control group of younger women precludes direct comparison of chromosomal abnormality rates between AMA and non-AMA pregnancies within our study.

Future research should prioritize multicenter or population-based studies in India to establish national benchmarks for chromosomal abnormality prevalence in AMA pregnancies. Larger datasets would also allow better analysis of age-specific risks and CNV spectra. Longitudinal follow-up of infants identified with VOUS or submicroscopic CNVs will be essential for clarifying the clinical relevance of such findings.

## 6 Conclusion

The present study provided regional evidence on the overall prevalence and spectrum of chromosomal abnormalities, which are observed among AMA pregnancies in Tamil Nadu, India. In addition, the findings confirmed the increased susceptibility associated with chromosomal abnormalities with advancing maternal age and demonstrated additional diagnostic contribution of CMA beyond the conventional karyotyping technique. Although these observations are found to be consistent with that of previous literature studies, this current investigation adds clinically relevant data from an Indian prenatal testing population and supports the integration of CMA into risk-based prenatal diagnostic pathways. In conclusion, the study presented that AMA pregnancies presented with a significant burden from the prenatal assessment on chromosomal abnormalities, with risks escalating sharply among women aged ≥40 years. Conventional karyotyping remained indispensable in the detection of common aneuploidies; however, CMA provides important incremental diagnostic yield for identifying clinically significant CNVs. The study findings underscored the need for tailored counseling approaches, potential utility of CMA for refining diagnostic accuracy, and importance of continued vigilance in prenatal care for AMA mothers.

## Data Availability

The datasets presented in this article are not readily available because the underlying study data are owned by LifeCell International Private Ltd. and are subject to institutional and confidentiality restrictions. Requests to access the datasets should be directed to the corresponding author and will be considered subject to the necessary institutional permissions.
